# Disk-based *k*-mer counting on a PC

**DOI:** 10.1186/1471-2105-14-160

**Published:** 2013-05-16

**Authors:** Sebastian Deorowicz, Agnieszka Debudaj-Grabysz, Szymon Grabowski

**Affiliations:** 1, Institute of Informatics, Silesian University of Technology, Akademicka 16, Gliwice, 44-100, Poland; 2, Institute of Computer Science, Lodz University of Technology, Al. Politechniki 11, Łódź, 90-924, Poland

**Keywords:** *k*-mer counting, de Bruijn graph genome assemblers, Multiple sequence alignment, Repeat detection

## Abstract

**Background:**

The *k*-mer counting problem, which is to build the histogram of occurrences of every *k*-symbol long substring in a given text, is important for many bioinformatics applications. They include developing de Bruijn graph genome assemblers, fast multiple sequence alignment and repeat detection.

**Results:**

We propose a simple, yet efficient, parallel disk-based algorithm for counting *k*-mers. Experiments show that it usually offers the fastest solution to the considered problem, while demanding a relatively small amount of memory. In particular, it is capable of counting the statistics for short-read human genome data, in input gzipped FASTQ file, in less than 40 minutes on a PC with 16 GB of RAM and 6 CPU cores, and for long-read human genome data in less than 70 minutes. On a more powerful machine, using 32 GB of RAM and 32 CPU cores, the tasks are accomplished in less than half the time. No other algorithm for most tested settings of this problem and mammalian-size data can accomplish this task in comparable time. Our solution also belongs to memory-frugal ones; most competitive algorithms cannot efficiently work on a PC with 16 GB of memory for such massive data.

**Conclusions:**

By making use of cheap disk space and exploiting CPU and I/O parallelism we propose a very competitive *k*-mer counting procedure, called KMC. Our results suggest that judicious resource management may allow to solve at least some bioinformatics problems with massive data on a commodity personal computer.

## Background

Counting the number of occurrences of every substring of length *k* (so-called *k*-mer) in a given string *S* is an important procedure in bioinformatics. One prominent application is *de novo* assembly from very large number (typically, a few billions) of short reads, produced by second-generation sequencing instruments. The most popular assembly approach for such data is based on building the de Bruijn graph [1], in which an edge between any pair of *k*-mers, represented as nodes in the graph, exists if and only if the (*k*−1)-symbol long suffix of one *k*-mer is a prefix of another. The current sequencing technology cannot, however, get rid of a relatively large number of errors (mis-detected nucleotides) in sequence reads. These errors can be detected on a statistical basis. The whole genome obtains high coverage (30-fold to 60-fold is typical) on modern sequencing platforms, which means that any “genuine” substring of length, e.g., 25 or 30 is very likely to appear multiple times in the reads collection. If a given *k*-mer occurs only once, it almost certainly contains at least one (or more probably a few) false symbol. The unique *k*-mers should be discarded before (or in the process of) building the de Bruijn graph, since they significantly increase the memory and time requirements for graph generation. Other applications of *k*-mer counting include fast multiple sequence alignment [2] and repeat detection [3]. Usually we should not distinguish between a *k*-mer and its reversed complement, and by the “canonical *k*-mer” we will mean the lexicographically smaller of the two.

Counting *k*-mers is challenging, because it should be both fast and performed using as little memory as possible. A naïve approach is to use a standard hash table, with *k*-mers as keys and their counts as values. This solution is both memory consuming and hard to parallelize effectively. Moreover, some of the *k*-mer counting tools cooperate with Quake [4], a widely used package to correct substitution sequencing errors in experiments with deep coverage, which takes *k*-mer statistics as an important component for its job. Supporting Quake makes *k*-mer counting even more demanding, both in time and used memory, since instead of plain *k*-mer counts Quake takes into account also the quality of base calls, i.e., high-quality reads have higher impact. In the following paragraphs we will briefly present several respected *k*-mer counting solutions.

Tallymer [3] is a *k*-mer counter based on the suffix array structure. Alas, suffix array construction is a relatively expensive operation, which is worsened for the second-generation sequencing data, where short reads must go together with high coverage.

Meryl, from the *k*-mer package of the Celera assembler [5], sorts the *k*-mers in memory. More precisely, it distributes all *k*-mers into (by default) 2^24^ bins and then sorts each bin. Finally it removes the unique ones. This approach requires a huge amount of memory to work. For example, for the human NA19238 dataset that we use in the experimental section (cf. the statistics in Table 1) over 350 GB of RAM would be needed, provided 8 bytes per *k*-mer.

**Table 1:** **Statistics of the datasets used in the experiments for*k=25***

	***D. ananassae***	***C. elegans***	***Z. mays***	***H. sapiens***	***H. sapiens***
				**NA19238**	**HG02057**
FASTQ file size [GB]	8.7	16.4	45.9	353	208
Total gzipped size [GB]	1.8	4.6	16.3	116.6	65.9
No. of gzipped files	6	2	108	463	6
No. of reads [ ×10^6^]	35	68	62	2,662	860
Read lengths	75	100	25–2043	36(most)–75	100
		**Statistics of *k *-mers**			
No. of singletons	43	347	1,010	11,823	3,367
No. of distinct	63	459	1,916	14,599	6,023
No. of distinct non-singletons	20	112	906	2,776	2,657
Total no.	1,803	5,127	20,214	44,687	65,325

Totals in millions.

Jellyfish [6] is an algorithm designed for shared memory parallel computers with multiple CPUs / cores. It uses several lock-free data structures. Efficient shared access to these structures implies high utilization of concurrent processing units. More precisely, Jellyfish is based on a hash table with quadratic probing collision resolution, where concurrent update operations are possible thanks to its lock-free mechanism, exploiting the ‘compare-and-swap’ (CAS) assembly instruction present in all modern multi-core CPUs. A CAS instruction performs up to three ‘elementary’ operations in an atomic fashion: it reads a memory cell, compares the read value to the second parameter of the instruction, and if the two values are equal it then writes the third CAS parameter to the memory cell. In the considered application this mechanism is much more efficient than traditional serialization of the access to a shared resource with locking. Another interesting feature of Jellyfish is to store only a part (prefix) of the *k*-mer in the hash table, since its suffix can be deduced from the hash position.

BFCounter [7] ingeniously involves the Bloom filter structure to discard most of the unique *k*-mers before their statistics are calculated using a standard hash table. Bloom filter (BF) is a classic compact probabilistic data structure for dynamic set membership queries, which allows a low rate of false positives. To count non-unique *k*-mers, BFCounter uses both a BF and a plain hash table. Initially, the *k*-mers are inserted into the BF structure. Then, the hash table is populated with all *k*-mers which have at least two occurrences plus a relatively small number of unique *k*-mers, those which appeared false positives in the BF. This algorithm is relatively frugal in memory utilization, but only a serial implementation exists.

It should be noted that both Jellyfish and BFCounter require estimation on the number of distinct *k*-mers. If the user-specified value is far from the real one, these algorithms may work much slower and using much more memory than in the case of appropriate values given. (More precisely, using Jellyfish with bounded memory is possible, but with limitations. This aspect is discussed in more detail in Sect. Results and discussion.)

We mention also khmer [8], a toolkit for *k*-mer-based dataset analysis, which (among others) can count *k*-mers and remove reads with low- or high-abundance *k*-mers. It is reasonably fast and memory frugal, but these benefits are achieved thanks to its probabilistic nature (again, due to the underlying Bloom filter): with a low probability, khmer may report a false *k*-mer as being “present”. Also the reported counts for genuine *k*-mers are only approximate. While these features are acceptable in some applications, we can name its drawbacks: no *k*-mer listing possibility (testing *every* possible *k*-mer is of course prohibitive, even for relatively small *k*) and no quality score support (e.g., with Quake-compatible counters). For these reasons, we do not compare khmer with our solution in the experimental section, as in our opinion they “play in different leagues”.

Very recently, a disk-based *k*-mer counting algorithm, named DSK, was presented [9]. On the high level, DSK is similar to the solution presented in this paper, but both algorithms were designed independently at about the same time. In DSK, the (multi)set of *k*-mers from the input reads is partitioned and partitions are sent to disk. Then, each partition is loaded and processed separately in the main memory, using a hash table. The tool provides strict control of the allocated memory and disk space (lower memory usage results in increased processing time due to more iterations and thus increased I/O), which for example allows to process human genome data in 4 GB of RAM only, in reasonable time.

## Methods

**Figure 1:**
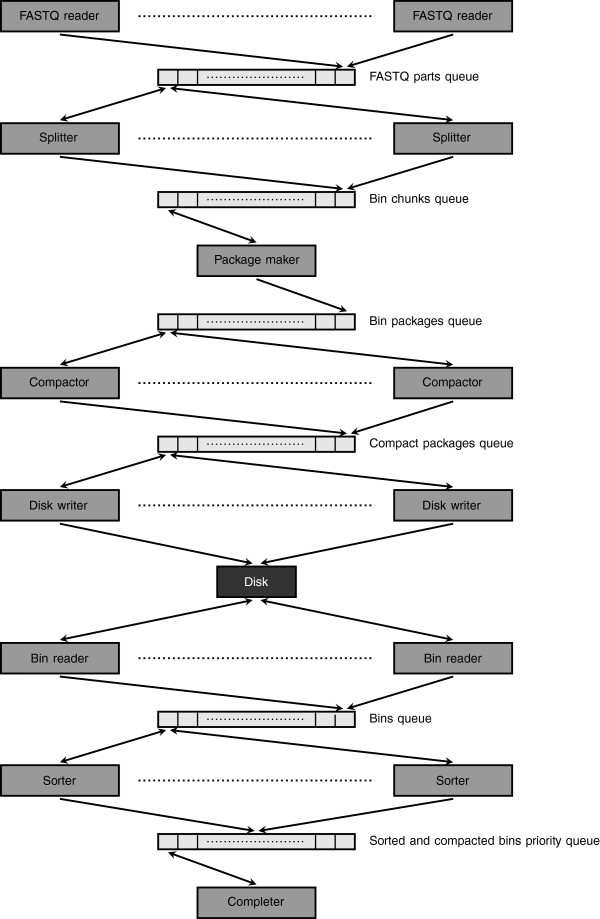
A scheme of the parallel KMC algorithm.

In the following subsections we first present our basic idea, on a high level and in a sequential manner, and then the ‘real’ parallel algorithm, involving multiple CPUs / cores and multiple disks (see Figure 1). The algorithm description is valid for any parameters *k* and read length *r*. In fact, in the current implementation *k* can be as large as 256, and *r* as large as 10240, provided that 10<*k*≤*r*. (These values can be easily increased when needed, as they are compile-time constants.)

We assume here that the purpose is to count the *k*-mers, but our implementation is more flexible: the associated quality scores can be taken into consideration, *k*-mers with a count below a threshold may be removed, etc. At least from the algorithmic point, however, the core functionality is the most important, hence it is discussed below.

### Basic idea

Our goal is to obtain a compact on-disk dictionary structure with *k*-mers as keys and their counts as values. The structure can then be read sequentially, or individual *k*-mers (with their associated counts) can be found using the standard binary search technique.^a^ The algorithm follows the disk-based distribution sort paradigm. In the first, distribution, phase, we scan the reads one by one, extract all the *k*-mers from each, replace them with their canonical versions when necessary, and send each to one of multiple (several hundred) disk files based on the *k*-mer prefix of length *p*_1_. Actually, the first phase starts with storing the data in buffers in the main memory where another prefix part, of length *p*_2_, is removed from each *k*-mer, and the prefix counts are maintained for further recovery. Once the buffer reaches the predefined capacity, its content is sent to a file.

The latter, sorting phase, is to collect the data from disk in the order of lexicographically sorted prefixes of length *p*_1_, recover the *p*_2_-symbol long prefixes, then radix-sort the *k*-mers, count their frequencies (after sorting repeating *k*-mers are at adjacent positions), and (optionally) remove, e.g., unique *k*-mers.

### Parallel algorithm

The detailed description of our algorithm is relatively complex and the reader is advised to consult Figure 1. The number of components (splitters, bin readers etc.) at each stage is chosen depending on the number of CPUs / cores of the target machine, but optionally these parameters may be user-specified. The distribution and the sort phase are described in the two following subsections.

#### Distribution phase

The first phase begins with reading blocks of several megabytes (the exact size depends on the available memory), rounded down to a record boundary, from a (possibly compressed) FASTQ dataset.^b^ One or more threads are used, depending on the number of input data files; the default number of such threads can be overridden with a command-line switch. The blocks are added to a queue.

In the next step, a number of splitter threads remove the blocks from the queue and extract *k*-mers from their reads, converting the *k*-mers to canonical form. Every splitter has its multiple (typically hundreds) bins to fulfill, each with capacity of, e.g., 2^15^ entries. Each *k*-mer is directed to the bin specified by the *k*-mer’s prefix of length *p*_1_.

**Figure 2:**
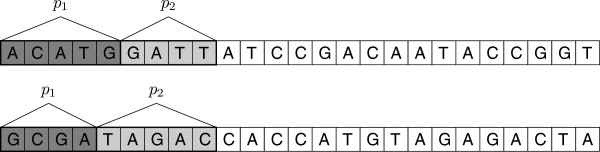
**An example of 2-stage *k*-mer prefix removal, for one *k*-mer starting with symbol A and one starting with non-A.** In total, 9 starting characters are effectively removed before storing the *k*-mer on a temporary disk. The part of length *p*_1_ stands for the ID of the bin the given *k*-mer is inserted into, and the part of length *p*_2_ is discarded to reduce the temporary storage (a way to recover later the removed part of length *p*_2_ is not shown here, for clarity; see text).

**Figure 3:**
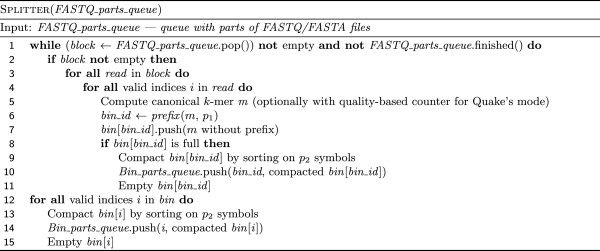
**Algorithm of splitting *k*-mers in reads to bins according to their prefix.** A full bin is compacted by sorting on *p*_2_-symbol long prefix and removing this prefix.

The lengths *p*_1_ are variable-sized and their minimum size is user-specified. For example, if the program is run with switch *-p3*, it means that 3, 4, or 5 symbols belong to the prefix, depending on its content. The rationale is that, for example, about 7/16 of all *canonicalk*-mers are those starting with A^c^, and different prefix lengths allow to have the bin counts more balanced. Due to some technical difficulties we resigned from even more granularity, but it should be stressed that this issue is practically irrelevant for the overall processing time. A more important goal is to limit the largest bin count (which is beyond our full control, since the reads content is not random). The number of resulting bins for parameter *-p3* is about 125, for *-p4* about 500, and for *-p5* about 2000. As a rule of thumb, it is better to use *-p5* for large collections (e.g., mammalian genomes with high coverage), *-p3* for small collections (e.g., bacterial genomes), and *-p4* for middle-sized ones. Once a bin is full, its content is transformed and then flushed to a common queue of bins. The transformation means here: partial sorting and compaction. The former uses counting sort, according to *k*-mer’s prefix, this time of the length *p*_2_. The latter operation, compaction, removes those prefixes and stores their frequencies, to enable to recover the *k*-mers later. The parameter *p*_2_ is chosen dynamically to fit other (possibly user-selected) settings, like *p*_1_, hardware configuration, and the values of *k* and *r*, with the idea of minimizing the amount of temporary data on disk (and thus also total I/O). For convenience (byte-aligned data layout) we always have *k*−*p*_1_−*p*_2_ a multiple of 4. The reader is advised to look at Figure 2 with a 2-stage prefix removal example presenting two cases: one for a *k*-mer starting with A and one for a *k*-mer starting with another symbol. The whole splitter operation is presented in Figure 3.

**Figure 4:**
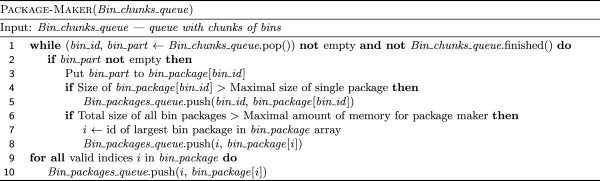
Algorithm of collecting compacted bin chunks from all splitting threads.

Now a single package maker thread comes into action. It prepares data to be sent to disk. More precisely, it moves the content from the queue of bins to another queue of “bin part packages” (Figure 4), which is handled by multiple threads. A compactor checks if it pays to strip extra 4 symbols from the prefix of each item in its package; the compaction criterion is satisfied if the prefix counters (statistics) together with the stripped data use less space than before the stripping. Now, the resulting data (possibly more compacted) are formed into one of many compact packages. Once the compact packages reach in total the specified maximum capacity, the largest of them is dumped to a file. Compacting the packages speeds up file handling and reduces file fragmentation. The *k*-mers scattered over (usually) hundreds of files are the outcome of the first, i.e., distribution phase. Each file corresponds to a unique prefix of length *p*_1_. In each file, the *k*-mers are also grouped by their successive *p*_2_ or *p*_2_+4 symbols. Assume for presentation clarity that the extra 4 symbols are not removed, and thus what is sent to files are (*k*−*p*_1_−*p*_2_)-symbol long suffixes of the *k*-mers, packed into bytes. Additionally, each file contains $4^{p_{2}}$ prefix counts (each stored in 2 bytes) which enable to recover the *p*_2_-symbol long parts of the prefixes. In total, the used disk space, in the worst case and with classic counters, is approximately

$$n_{k}(k-p_{1}-p_{2})/4+2\cdot 4^{p_{2}}\cdot n_{k}/2^{15}\text{bytes,}$$

where *n*_*k*_=*n*(*r*−*k*+1) is the number of *k*-mers in the input data. Switching to Quake-compatible counters increases this worst-case estimate to

$$n_{k}(k-p_{1}-p_{2}+16)/4+2\cdot 4^{p_{2}}\cdot n_{k}/2^{15}\text{bytes.}$$

In practice, removing the prefixes reduces the disk usage by at least a few, and sometimes over 20 percent, depending on the value of *k*. This has an analogous effect of reducing the I/O, which translates to similar overall performance improvement.

#### Sorting phase

The second phase starts with bin reader threads; there are as many of them as disks for temporary data. The bin readers read the files from disk to a queue.

Now, several sorter threads collect the data from the queue, uncompact the *k*-mers (their *p*_2_-symbol long prefix parts are brought back), sort them using multithreaded least significant digit (LSD) radix sort (the sort implementation is partially inspired by [10]), count their frequencies and discard *k*-mers with out of thresholds counter values (based on default or user-selected settings). The input data to be sorted are divided evenly among threads.

**Figure 5:**
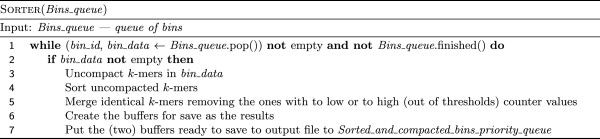
**Algorithm of sorting *k*-mers within bins.** Fast radix sort procedure is used here.

The (remaining) *k*-mers, with their counts, are ready to be sent to disk (cf. Figure 5), but it is up to the next stage, the completer thread. The sorter threads submit the *k*-mers with their statistics into a priority queue, with bin ID as the priority, which is then handled by the single completer thread. This thread reorganizes the sorted bins in the order of ascending bin ID. The priority queue is needed to send the statistics in the proper order. As this structure is relatively small, implementing it as a plain unsorted array with several hundred slots and linear scan for finding the minimum was enough for the application.

The scheme presented above depends on a number of parameters. By default, KMC works in an automatic mode, where the parameters are found with respect to the machine it is executed at; the number of CPU cores and the available amount of RAM are all taken into account. More details on the parameter setting are given in Additional file 1: Table S6). (Manual setting is possible as well, though.)

## Results and discussion

Our algorithm, called K-mer Counter (KMC), was implemented in C++11, using gcc compiler (version 4.7.1) for the linux build and Microsoft Visual Studio 2012 for the Windows build, and is freely available at http://sun.aei.polsl.pl/kmc (as well as is given as Additional files 2 and 3). The following well-known libraries were used: OpenMP, Boost for managing the threads and filesystem operations, zlib and libbzip for reading compressed FASTQ files, and asmlib (http://www.agner.org/optimize/asmlib-instructions.pdf) for a fast low-level memcpy^d^ implementation.

Out of the five well-known *k*-mer counting tools, three were taken for the comparative tests, Jellyfish [6], BFCounter [7], and DSK [9]. The other two, Tallymer [3] and Meryl from the Celera assembler [5], were tested in [6], on a 1 GB turkey genome, and we can find the following statement in the cited work: *Jellyfish is also able to count 22-mers at coverage > 10× where the other programs fail or take over 5 h.* Clearly, this makes them hard to use on human genome data, with 30-fold coverage.

Two test machines were used. One was a 4 AMD Opteron™6136 2.4 GHz CPUs (32 cores) server with 128 GB RAM, and fast RAID-0 disk matrix of total size 2.0 TB. The other was a “home” PC, with 6-core AMD Phenom II 1090 3.2 GHz CPU, 16 GB RAM and 3 SATA HDD of sizes 2 TB each. The hard disks at the PC machine were: two Seagate Barracuda Green ST2000DL003 with 5,900 rpm and one Hitachi Deskstar 7K3000 with 7,200 rpm.

The comparison includes total computation time and maximum RAM use. Moreover, the maximum disk use of the disk-based algorithms is recorded. Although the other tested programs for *k*-mer counting, except for DSK, work only in RAM, we believe that using even several hundreds of gigabytes of disk space during the execution of KMC is a mild price for the achieved high efficiency, as disk space is almost two orders of magnitude cheaper than the RAM memory. KMC was run twelve times for each dataset and each tested *k*: with 32 GB and 16 GB RAM limitation on the server machine and with 11 GB RAM limitation on the PC, with classic and Quake-compatible counters, and with gzipped and non-compressed input data in each configuration. The classic counters are just integers telling how many times a *k*-mer occurs in the dataset. The Quake-compatible counters take into account the quality scores and are thus decimal-point numbers. The other programs used in the comparison, except DSK, optionally produce output results in Quake-compatible form.

We performed experiments on five datasets, three of which are presented below and an extra two in Additional file 1.

These three datasets discussed here comprise: 

•*Homo sapiens* NA19238 from 1000GP (http://ftp.1000genomes.ebi.ac.uk/vol1/ftp/data/NA19238/sequence_read/), used also in [7],

•*Homo sapiens* HG02057 from 1000GP (http://ftp.1000genomes.ebi.ac.uk/vol1/ftp/data/HG02057/sequence_read/),

•*Caenorhabditis elegans* (http://ftp.sra.ebi.ac.uk/vol1/fastq/SRR065/SRR065390/).

Their basic statistics, for *k*=25, are presented in Table 1.

There were several problems to compare exactly the proposed algorithm against its competitors. For example, some datasets contain raw reads with occasional N symbols in the DNA stream. Jellyfish processes such reads but refrains from counting the *k*-mers containing Ns (in KMC we handle this issue in the same way). To our knowledge, BFCounter treats Ns as As and DSK treats them as Gs. This is of course rather strange from biological point of view but since there are only very few Ns, the misinterpreted *k*-mers do not affect noticeably the RAM occupancy and computation time. Another problem with BFCounter is that it fails when executed with *k*>25 in the classic counters mode (the authors claim it should work for *k* up to 31, but we were not able to reproduce it). With the Quake-compatible counters it sometimes handles larger *k*, sometimes fails (details in the tables).

KMC is clearly the most flexible software: it can work over wide ranges of *k* and also for both counter modes. Like DSK, but contrary to other solutions, KMC can also read gzipped FASTQ datasets (typically used in genomic repositories) which tends to improve overall processing time (due to reduced I/O and the possibility to read multiple gzip files, even located at multiple disks). It is important to note that KMC space resources are bounded: the RAM usage is user-selected and the upper bound on the amount of disk space can be calculated with the (approximate) formula given in Section Distribution phase, which depends only on standard input parameters (the number of reads, the read length, the value of *k*). DSK is even better in this aspect: it allows to set the RAM and disk usage quite precisely, but of course choosing tight parameters comes at a price of increased I/O and thus overall processing time. On the other hand, BFCounter and Jellyfish require guessing the number of unique *k*-mers in the dataset, and a significant deviation from the true value is likely to cost significantly in increased RAM usage and processing time. In fact, it is possible to bound (not very precisely) the RAM requirements for Jellyfish, which, for large enough data, results in two-stage processing. In the first stage the tool produces several hash tables, and then, in the second stage, it merges them. The price is, however, deterioration in speed. Moreover, this regime of work is viable only for classic counters, as for the Quake-compatible counters the amount of space Jellyfish needs in the second stage is huge and we were not able to test it on human datasets.

Based on the results, several observations can be made. We start from the two human datasets. The first, NA19238^e^ (Table 2), has variable-length reads, but most of them are short, of length 36 only. This fact poses a restriction on the maximum used value of *k* (31). BFCounter was many times slower than its competitors and the amount of RAM it used was not impressive either: up to 46 GB, i.e., less than Jellyfish (run with speed in mind, i.e., with possibly the smallest amount of allocated memory for which no hash table merging is needed) but more than KMC. KMC was consistently faster than Jellyfish (both with classic and Quake-compatible counters) and several times faster than DSK. On the other hand, DSK (run in this and all other tests with default settings) was clearly the most frugal in memory use (6 GB) but required about twice or more disk space than KMC. When the KMC’s input FASTQ data are gzipped (note that the input data consist of 463 gzipped files and several of them can be read in parallel), the gap in speed between KMC and Jellyfish sometimes exceeds factor 2. Although the speedup thanks to compressed input varies, it is often of the order of 20 percent or more. The amount of disk space needed by KMC is up to 141 GB in the classic counters mode and up to 321 GB with the Quake-compatible counters. While certainly not small, this is less than the size of the input (uncompressed) FASTQ file. Reducing the amount of available RAM from 32 GB to 16 GB for KMC results in about 10 percent slow-down.

**Table 2:** ***k*-mers counting results for*Homo sapiens* NA19238 individual (353 GB FASTQ file or 463 gzipped FASTQ files of total size 116.6 GB)**

	***k=22***		***k=25***		***k=28***		***k=31***	
**Algorithm**	**Space**	**Time**	**Space**	**Time**	**Space**	**Time**	**Space**	**Time**
	**Classic counters**							
	32-core server							
BFCounter	46/ 0	114,083	41/ 0	99,468	Failed		Failed	
Jellyfish	50/ 0	2,303	64/ 0	2,258	75/ 0	2,208	87/ 0	2,107
Jellyfish^1^	27/ 39	2,964	33/ 36	2,769	21/ 27	2,673	24/ 22	2,511
DSK	6/200	6,490	6/340	6,020	6/280	5,115	6/221	4,215
DSK ^*gz*^	6/200	9,076	6/340	8,029	6/280	7,157	6/221	6,424
KMC	32/104	1,405	32/130	1,488	32/133	1,522	32/121	1,471
KMC	16/107	1,548	16/131	1,657	16/141	1,684	16/128	1,568
KMC ^*gz*^	32/104	1,040	32/129	1,066	32/132	1,055	32/120	989
KMC ^*gz*^	16/107	1,278	16/130	1,631	16/141	1,662	16/125	1,307
	6-core PC							
DSK	6/200	21,468	6/340	18,774	6/280	15,384	6/221	11,857
DSK ^*gz*^	6/200	18,939	6/340	16,818	6/280	14,694	6/221	12,070
KMC	11/107	3,482	11/128	3,442	11/138	3,584	11/127	3,515
KMC ^*gz*^	11/107	2,198	11/128	2,206	11/138	2,303	11/127	2,365
	**Quake-compatible counters**							
	32-core server							
BFCounter	70/ 0	171,888	72/ 0	180,861	Failed		Failed	
Jellyfish	100/ 0	4,339	57/230	2,891 ^∗^	64/192	3,246 ^∗^	70/175	3,161 ^∗^
KMC	32/311	2,585	32/302	2,467	32/282	2,347	32/237	2,106
KMC	16/315	2,615	16/305	2,730	16/283	2,592	16/245	2,284
KMC ^*gz*^	32/310	2,071	32/302	1,995	32/282	1,880	32/237	1,690
KMC ^*gz*^	16/318	2,273	16/304	2,611	16/283	2,015	16/244	2,707
	6-core PC							
KMC	11/316	5,538	11/298	5,533	11/277	5,184	11/242	5,016
KMC ^*gz*^	11/316	5,370	11/298	5,060	11/277	4,708	11/243	4,643

RAM and disk spaces (the first and the second value in the column “Space”, respectively) are in GB (1GB= 2^30^B). Time is in seconds. The test machines: 32-core server, 6-core PC (see more details in the text). Superscripts denote: ^1^—RAM limited to 36GB, ^*gz*^—input data in gzipped files. The programs were used for the number of threads adjusted to the number of cores to achieve maximum speed. The asterisk signs (for Jellyfish) denote that two separate databases were constructed by Jellyfish due to the memory limit of the machine (128GB RAM) and Jellyfish reported that to merge these databases it needs more RAM, so these times are underestimated.

The HG02057 data (Table 3) are quite challenging, concerning their sheer volume. BFCounter was not tested here, since the experiments would take literally weeks. Jellyfish suffers from rapidly growing RAM usage for larger *k*, and for *k*=31 it needs 86 GB of memory. On the other hand, KMC can handle the dataset in 32 GB or even 16 GB of RAM, no matter the *k*. In the runs with halved memory, KMC is (again) only by about 10 percent slower than with 32 GB, and requires a few percent more disk space. We admit that Jellyfish is usually faster on this dataset than KMC-32 GB, by about 10–20 percent. The penalty in RAM usage is severe though; in the Quake-compatible mode Jellyfish couldn’t properly finish some runs on the server machine with 128 GB of RAM, i.e., produced two output files and was unable to merge them in the available memory (the corresponding results are marked with an asterisk). The analogous weakness of KMC, using up to 553 GB of temporary disk space, is less painful (not only disk space is much cheaper than internal memory, but also adding a disk to the system is usually easier and not so limited as expanding RAM). We note that Jellyfish’s problems with memory are partly related to its dependence on the estimate of the number of unique *k*-mers. Jellyfish works fastest when the whole hash table it needs fits the RAM memory. Alas, it requires knowing (approximately) the number of unique *k*-mers. If the specified parameter is too small, Jellyfish creates several temporary files on disk, which are at the end merged; an operation not only time-consuming, but also demanding in memory, as our experiment showed. Again, for this dataset DSK may be a tool of choice on a less powerful (e.g., laptop) computer, since it uses only 6 GB of RAM and usually less disk space than KMC (which is at least 4 times faster though).

**Table 3:** ***k*-mers counting results for*Homo sapiens* HG02057 individual (208 GB FASTQ file or 6 gzipped FASTQ files of total size 65.9 GB)**

	***k=22***		***k=28***		***k=40***		***k=55***	
**Algorithm**	**Space**	**Time**	**Space**	**Time**	**Space**	**Time**	**Space**	**Time**
	**Classic counters**							
	32-core server							
Jellyfish	27/ 0	1,375	75/ 0	1,433	—		—	
Jellyfish^1^	27/ 0	1,375	21/ 53	2,404	—		—	
DSK	6/168	8,683	6/156	9,073	6/195	13,172	6/197	9,409
DSK ^*gz*^	6/168	10,125	6/156	10,579	6/195	14,569	6/197	10,987
KMC	32/130	1,221	32/220	1,706	32/341	2,486	32/391	2,722
KMC	16/134	1,376	16/234	1,872	16/343	2,664	16/405	2,967
KMC ^*gz*^	32/130	1,249	32/219	1,505	32/342	2,304	32/391	2,597
KMC ^*gz*^	16/134	1,195	16/234	1,732	16/343	2,479	16/403	2,909
	6-core PC							
DSK	6/168	22,963	6/156	23,512	6/195	37,958	6/197	28,681
DSK ^*gz*^	6/168	21,688	6/156	22,061	6/195	36,328	6/197	26,584
KMC	11/137	2,939	11/234	3,782	11/343	6,133	11/405	7,770
KMC ^*gz*^	11/136	2,623	11/235	4,041	11/343	6,306	11/405	7,020
	**Quake-compatible counters**							
	32-core server							
Jellyfish	51/ 0	2,426	59/126	2,503 ^∗^	—		—	
KMC	32/388	2,612	32/468	3,011	32/537	3,541	32/542	3,546
KMC	16/402	2,990	16/468	3,405	16/539	4,300	16/552	4,175
KMC ^*gz*^	32/387	2,409	32/468	2,860	32/537	3,370	32/536	3,357
KMC ^*gz*^	16/400	2,760	16/468	3,285	16/498	4,083	16/552	4,038
	6-core PC							
KMC	11/404	6,625	11/469	7,741	11/539	9,673	11/552	11,135
KMC ^*gz*^	11/403	6,783	11/468	8,034	11/539	9,764	11/553	9,775

Test methodology and column description are just as for Table 2. The asterisk sign (for Jellyfish) denotes that two separate databases were constructed by Jellyfish due to the memory limit of the machine (128GB RAM) and Jellyfish reported that to merge these databases it needs more RAM, so these times are underestimated.

On the smallest of the tested datasets, *C. elegans* (Table 4), our tool, KMC, cannot fully spread its wings and achieve better results than Jellyfish. In the amount of used RAM memory they are comparable, with 21–26 GB used by Jellyfish, slightly growing with chosen *k*, and (selectable) 16 GB or 32 GB spent by KMC. In speed, Jellyfish was in most cases faster by 10–30 percent than KMC (but the speed varied somewhat; for more results see Additional file 1: Table S2). BFCounter used here the least amount of RAM memory (4 GB) but was about 40 times slower than KMC and Jellyfish. DSK used 5 GB of RAM and less disk space than KMC, but was a few times slower than KMC and Jellyfish.

**Table 4:** ***k*-mers counting results for*Caenorhabditis elegans* (16.4 GB FASTQ file or 2 gzipped FASTQ files of total size 4.6 GB)**

	***k=22***		***k=28***		***k=40***		***k=55***	
**Algorithm**	**Space**	**Time**	**Space**	**Time**	**Space**	**Time**	**Space**	**Time**
	**Classic counters**							
	32-core server							
BFCounter	4/ 0	10,407	Failed		Failed		Failed	
Jellyfish	21/ 0	88	22/ 0	89	—		—	
DSK	5/13	493	5/13	458	5/16	647	5/16	481
DSK ^*gz*^	5/13	587	5/13	564	5/16	761	5/16	592
KMC	32/10	105	32/18	115	32/27	185	32/31	191
KMC	16/11	93	16/18	93	16/27	163	16/32	160
KMC ^*gz*^	32/10	134	32/18	138	32/27	199	32/31	203
KMC ^*gz*^	16/11	119	16/18	121	16/27	195	16/32	182
	6-core PC							
DSK	5/13	1,307	5/13	1,215	5/16	2,073	5/16	1,597
DSK ^*gz*^	5/13	1,268	5/13	1,151	5/16	2,017	5/16	1,518
KMC	11/11	274	11/18	343	11/27	507	11/32	553
KMC ^*gz*^	11/11	233	11/18	333	11/27	514	11/32	542
	**Quake-compatible counters**							
	32-core server							
BFCounter	4/ 0	10,349	4/ 0	9,527	4/ 0	8,213	4/ 0	6,689
Jellyfish	24/ 0	143	25/ 0	132	—		—	
KMC	32/31	173	32/37	179	32/42	245	32/43	243
KMC	16/32	166	16/37	154	16/43	212	16/44	214
KMC ^*gz*^	32/31	184	32/37	188	32/43	247	32/43	249
KMC ^*gz*^	16/32	168	16/37	165	16/43	218	16/44	230
	6-core PC							
KMC	11/32	562	11/37	635	11/43	750	11/44	784
KMC ^*gz*^	11/32	555	11/37	627	11/43	749	11/44	772

**Figure 6:**
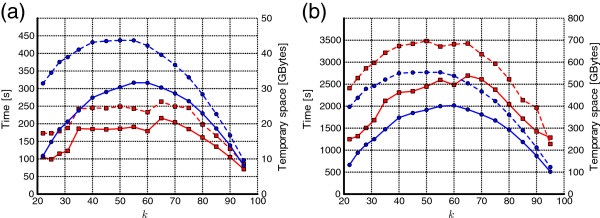
**The KMC processing time (red lines) and used disk space (blue lines) as a function of k for (a) C. elegans dataset, (b) HG02057 dataset.** The solid lines are for classic counters, the dashed lines for Quake-compatible counters.

As expected, KMC uses more time and disk space in the Quake-compatible counter mode, but in most cases these differences are by a factor smaller than 2. With growing *k*, the computational resources increase (until *k* becomes quite close to the read length), in accordance to the pattern demonstrated in Figure 6.

The comparisons with other tools were run on our stronger machine, but KMC is also tested on a PC, where 11 GB of RAM was always used. It is about 3 times slower and uses a comparable amount of disk space.

Figure 6 presents the computation time (red lines) and the used disk space (blue lines) for *C. elegans* and HG02057, for varying *k*. The read length *r* for both datasets is 100. On the charts, the solid lines are for the occurrence count mode and the dashed lines for the Quake-compatible mode. The statistics were gathered for counts at least 2 in the former and at least 2.0 in the latter mode. We focus on the case of *C. elegans* (a), although similar observations pertain to the other dataset. Not surprisingly, the Quake-related mode is more demanding in computation time and disk space, but the relative gap diminishes with growing *k*. The time grows suddenly when *k* exceeds thresholds 32 and 64, as more machine words are then needed to store a whole *k*-mer. As *k* was approaching *r*, KMC worked faster and in less space, because the number of *k*-mers per read was diminishing relatively fast. Another clear observation is that the processing time and used disk space are closely related; more precisely, the overall time and the amount of I/O are closely related in our algorithm.

**Figure 7:**
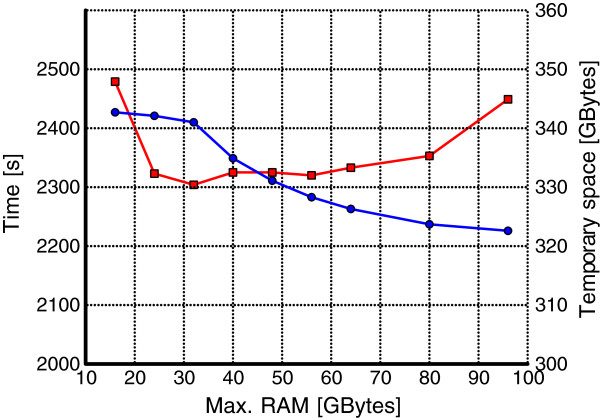
**HG02057 (gzipped) dataset, *k=40 *, classic counters.** The KMC processing time (red line) and used disk space (blue line) as a function of allowed RAM memory.

It may also be an interesting question how the processing time and the amount of used disk space in KMC vary with different specified amounts of RAM (i.e., if it pays to give it more RAM when it is available). The results, presented in Figure 7, concern the HG02057 dataset in gzipped input representation, for *k*=40 and in the classic counters mode. Using more than 32 GB of RAM is even detrimental for the KMC speed, although the loss is slight up to 80 GB (becomes somewhat more significant with 96 GB). What is perhaps more important practically, using 24 GB of RAM is almost as good as 32 GB. Another observation is that more RAM translates into less disk space needed, but this effect is mild (about 6 percent difference between the extreme values).

**Figure 8:**
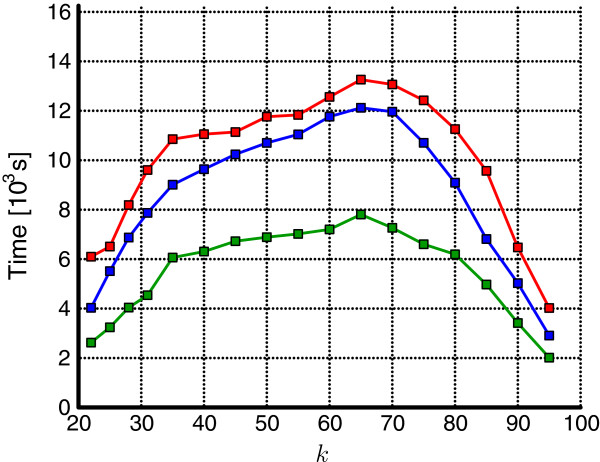
**HG02057 (gzipped) dataset, classic counters.** Influence of the no. of HDDs on the processing time: 3 HDDs (green line), 2 HDDs (blue line), and 1 HDD (red line).

A related experiment, on the same dataset, concerned the impact of the number of hard disks available in the PC test machine (Figure 8). While finding a precise formula would be difficult and dependent on many factors (gzipped or non-gzipped input, standard or Quake-compatible counters, etc.), this experiment clearly shows that using more than one disk is beneficial, and 3 disks reduce the overall processing time sometimes by even more than 50% compared to a single disk. This observation confirms that the overall KMC performance strongly depends on the I/O (sub)system and supplying the platform with SSD disk(s), with approximately 3 times faster transfer rates and 2 orders of magnitude shorter access times, should give an extra boost.

The architecture of KMC fits quite well the MapReduce (MR) paradigm [11], widely used in (but not limited to) cloud computing. Using this framework directly would be inefficient for the *k*-mer counting problem, due to enormous I/O and communication costs. In KMC the *k*-mer counting threads make use of the available RAM memory, and once their buffers are filled up, they sent statistics onto disk. Hence, these threads resemble the Combiner function in MR, which typically digests (“reduces”) the data produced by the Map function and outputs them to intermediate file(s). The *k*-mer statistics from disk are read and processed by other, merging, threads, making an analogy to the Reduce function in MR.

## Conclusion

High utilization of available resources is the key to obtaining competitive algorithms. Even home computers, equipped with multi-core CPUs, several gigabytes of RAM and a few fast TB-scale hard disks get powerful enough to be applied for real bioinformatics tasks. Our *k*-mer counter, KMC, being an external and parallel algorithm, is capable to find exact *k*-mer statistics on short-read human genomic collection with about 30-fold coverage in less than 40 minutes on a standard 6-core PC with 16 GB of RAM and 3 hard disks, and on long-read human genomic collection with a similar coverage in less than 70 minutes, for *k*=28 in both tests. Using a more powerful machine reduces the times more than twice. Even in a demanding scenario (Quake-compatible counters, *k*=70) our software works in less than 3 hours on the PC.

## Endnotes

^a^These functionalities of our tool are available via an API, whose detailed description is contained in Additional file 1.

^b^Our tool handles both FASTQ and FASTA input files. For brevity, we however refer throughout the paper only to the (more complicated of the two) FASTQ format.

^c^About 1/4 of all *k*-mers start with A and also about 1/4 of all *k*-mers end with T, and it is easy to note that for these (and only these) *k*-mers their canonical forms start with A. These two groups are not disjoint; their intersection, with exactly the *k*-mers having A as their first and T as their last symbol, contains about 1/16 of all *k*-mers. Taking all this into account we immediately obtain the figure 7/16. Similarly, we can show that the distribution of *k*-mers starting with base C, G and T is 5/16, 3/16 and 1/16. These numbers are different, since (roughly speaking) the lexicographically greater the first *k*-mer’s symbol, the lesser chance its canonical form will also start with the same symbol.

^d^memcpy is a popular function from the C language standard library, which copies a number of bytes from one memory location to another.

^e^Used in the experiments in [7], under a mistaken name NA19240.

## Competing interests

The authors declare no competing interests whatsoever.

## Authors’ contributions

SD developed the main concept of the algorithm, was the main architect of the KMC software, wrote most of the implementation code and ran the experiments; he also participated in drafting the manuscript. ADG adapted the parallel radix sort algorithm for the *k*-mer sorting, implemented the parallel scheme of the main algorithm and the API; she also assisted in drafting the manuscript. SG was a co-author of the main idea of sorting the *k*-mers and proposed the idea of compacting *k*-mers stored in temporary files; he was the main author of the text. All authors read and approved the final manuscript.

## Supplementary Material

Additional file 1 1) KMC counter usage. 2) API. 3) Example of API usage. 4) Database format. 5) Experimental results. 6) Automatic setting of parameters in KMC. 7) Selected components of the KMC algorithm (codes not shown in the main part of the paper). Click here for file

Additional file 2 Source codes for 64-bit Windows platform (Microsoft Visual C++ 2012 project). Click here for file

Additional file 3 Source codes for 64-bit Linux platform (gcc project). Click here for file
