# Supplementary material for: Disk-based k-mer counting on a PC
Source: BMC Bioinformatics. 2013 May 16;14:160. doi: 10.1186/1471-2105-14-160 (PMC3680041; doi:10.1186/1471-2105-14-160)
Supplement: Additional file 1 — 1) KMC counter usage. 2) API. 3) Example of API usage. 4) Database format. 5) Experimental results. 6) Automatic setting of parameters in KMC. 7) Selected components of the KMC algorithm (codes not shown in the main part of the paper). [file 1471-2105-14-160-S1.pdf]

Supplementary material for the paper

Disk-based k-mer counting on a PC

by

Sebastian Deorowicz, Agnieszka Debudaj-Grabysz, and Szymon Grabowski

Contents

1 KMC usage **2**

2 API **4**
2.1 CKmerAPI class 4
2.2 CKMCFile class 4

3 Example of API usage **6**

4 Database format **9**
4.1 The .kmc_pre file structure 9
4.2 The.kmc_suf file structure 10

5 Experimental results **11**
5.1 Test platforms 11
5.2 Parameters of programs 11
5.3 Results 12

6 Automatic setting of parameters in KMC **18**

7 Selected components of the KMC algorithm (codes not shown in the main part of the paper) **19**

1 KMC usage

KMC program constructs a database of statistics for input set of FASTQ files. This database can then be used from other software: directly via KMC API (described in Section 2) or by reading a textual file containing a list of k-mers and their related counters. This textual file can be obtained for a database by KMC-dump program, that is presented in Section 3 as a sample application of our KMC API. Section 4 describes the database format in detail for those interested in the low-level access to the data. Section 5 contains additional experimental results and a description of the parameters of execution of the examined programs. Section 6 contains description of how the automatic setting of parameters of KMC works.

Below we describe in detail the parameters and options of the KMC command-line tool, in version 0.3.

The general syntax is:

kmc [options] <input_file_name> <output_file_name> <working_directory> [<working_directory2> ...]

or:

kmc [options] <@input_file_names> <output_file_name> <working_directory> [<working_directory2> ...]

where the parameters are:

- input_file_name — a single file in FASTQ format (gzipped or not),
- @input_file_names — a file name with list of input files in FASTQ format (gzipped or not),
- output_file_name — the output database file; if such a file exists, it will be overwritten.

The configuration options comprise:

- -v — verbose mode (shows all parameter settings); default: false,
- -k<len> — k-mer length, k from 1 to MAX_K; default: 25,
- -m<size> — max amount of RAM in GB (from 4 to 1024); default: 32,
- -p<par> — set level of distribution (from 3 to 5); default: 4,
- -f[a/q] — input in FASTA format (-fa) or FASTQ format (-fq); default: FASTQ,
- -q[value] — use Quake's compatible counting with [value] representing lowest quality; default: 33,
- -ci<value> — exclude k-mers appearing less than <value> times; default: 2,
- -cs<value> — maximal value of a counter; default: 255,
- -cx<value> — exclude k-mers appearing more of than <value> times; default: 1e9,
- -b — turn off transformation of k-mers into canonical form,
- -sc<value> — number of compacting threads (if not specified, it is set automatically based on the total number of computation threads, see the previous parameter),
- -sf<value> — number of FASTQ reading threads,
- -sp<value> — number of splitting threads,
- -so<value> — number of sorter threads,

- -sr<value> — number of threads per single sorter.

The parameters -sc<value>, -sf<value>, -sp<value>, -so<value>, and -sr<value> concern the internal work of KMC, i.e., their settings may affect the program's processing speed, but won't change its output. Not setting at least one parameter from this group makes KMC ignore them all.

Here are some usage examples.

kmc -k27 -m24 NA19238.fastq NA.res \data\kmc_tmp_dir\

kmc -k27 -q -m24 @files.lst NA.res \data\kmc_tmp_dir\

2 API

In this section we describe two classes, CKmerAPI and CKMCFile. They can be used to gain access to the databases produced by KMC program.

2.1 CKmerAPI class

This class represents a k-mer. Its key methods are:

- CKmerAPI(uint32 length = 0) — constructor, that creates the array kmer_data of appropriate size,
- CKmerAPI(const CKmerAPI &kmer) — copy constructor,
- char get_symbol(unsigned int pos) — returns k-mer's symbol at a given position (0-based),
- std::string to_string() — converts k-mer to string, using the alphabet ACGT,
- void to_string(char *str) — converts k-mer to string, using the alphabet ACGT; the function assumes that enough memory was allocated,
- void to_string(str::string &str) — converts k-mer to string, using the alphabet ACGT,
- bool from_string(std::string &str) — converts string (from alphabet ACGT) to k-mer,
- CKmerAPI() — destructor, releases the content of kmer_data array,
- overloaded operators: =, ==, <.

2.2 CKMCFile class

This class handles a k-mer database. Its key methods are:

- CKMCFile() — constructor,
- bool OpenForRA(std::string file_name) — opens two files: file_name with added extension ".kmc_pre" and ".kmc_suf", reads their whole content to enable random access (in memory), and then closes them,
- bool OpenForListing(std::string file_name) — opens the file file_name with added extension ".kmc_pre" and allows to read the k-mers one by one (whole database is not loaded into memory),
- bool ReadNextKmer(CKmerAPI &kmer, float &count) — reads next k-mer to kmer and updates its count; the return value is bool; true as long as not eof-of-file (available only when database is opened in listing mode),
- bool Close() — if the file was opened for random access, the allocated memory for its content is released; if the file was opened for listing, the allocated memory for its content is released and the ".kmer" file is closed,
- bool SetMinCount(uint32 x) — set the minimum counter value for k-mers; if a k-mer has count below x, it is treated as non-existent,
- uint32 GetMinCount(void) — returns the value (uint32) set with SetMinCount,

- bool SetMaxCount(uint32 x) — set the maximum counter value for k-mers; if a k-mer has count above x, it is treated as non-existent,
- uint32 GetMaxCount(void) — returns the value (uint32) set with SetMaxCount,
- uint64 KmerCount(void) — returns the number of k-mers in the database (available only for databases opened in random access mode),
- uint32 KmerLength(void) — returns the k-mer length in the database (available only for databases opened in random access mode),
- bool RestartListing(void) — sets the cursor for listing k-mers from the beginning of the file (available only for databases opened in listing mode). The method OpenForListing(std::string file_name) invokes it automatically, but it can be also called by a user,
- bool Eof(void) — returns true if all k-mers have been listed,
- bool CheckKmer(CKmerAPI &kmer, float &count) — returns true if kmer exists in the database and set its count if the answer is positive (available only for databases opened in random access mode),
- bool IsKmer(CKmerAPI &kmer) — returns true if kmer exists (available only for databases opened in random access mode),
- void ResetMinMaxCounts(void) — sets min_count and max_count to the values read from the database,
- bool Info(uint32 &_kmer_length, uint32 &_mode, uint32 &_counter_size, uint32 &_lut_prefix_length, uint32 &_min_count, uint32 &_max_count, uint64 &_total_kmers) — gets current parameters from the k-mer database,
- CKMCFile() — destructor.

3 Example of API usage

The KMC-dump application (Figs. S1 and S2) shows how to list and print k-mers with at least min_count and at most max_count occurrences in the database. Fig. S1 presents parsing the command-line parameters, including -ci<value> and -cx<value>. Input and output file names are also expected. The code in Fig. S2 is for actual database handling. This database is represented by a CKMCFile object, which opens an input file for k-mer listing (the method bool OpenForListing(std::string file_name) is invoked). The parameter of the method SetMinCount (SetMaxCount) must be not smaller (not greater) than the corresponding parameter -ci (-cx) with which KMC was invoked (otherwise, nothing will be listed). The listed k-mers are in the form like:
AAACACCGT\t<value>
where the first part is the k-mer in natural representation, which is followed by a tab character, and its associated value (integer or float). (Such format is compatible with Quake, a widely used tool for sequencing error correction.) Note that, if needed, one can easily modify the output format, changing the lines 39 and 41 in Fig. S2.

```
#include <iostream>
#include "../kmc_api/kmc_file.h"

void print_info(void);

int _tmain(int argc, char* argv[])
{
  CKMCFile kmer_database;
  int i;
  uint32 min_count_to_set = 0;
  uint32 max_count_to_set = 0;
  std::string input_file_name;
  std::string output_file_name;

  FILE * out_file;
  //-------------------------------------------------------------
  // Parse input parameters
  //-------------------------------------------------------------
  if(argc < 3)
  {
    print_info();
    return EXIT_FAILURE;
  }

  for(i = 1; i < argc; ++i)
  {
    if(argv[i][0] == '-')
    {
      if(strncmp(argv[i], "-ci", 3) == 0)
        min_count_to_set = atoi(&argv[i][3]);
      else if(strncmp(argv[i], "-cx", 3) == 0)
          max_count_to_set = atoi(&argv[i][3]);
    }
    else
      break;
  }

  if(argc - i < 2)
  {
    print_info();
    return EXIT_FAILURE;
  }

  input_file_name = std::string(argv[i++]);
  output_file_name = std::string(argv[i]);

  if((out_file = fopen(output_file_name.c_str(),"w")) == NULL)
  {
    print_info();
    return EXIT_FAILURE;
  }

  setvbuf(out_file, NULL ,_IOFBF, 1 << 24);

  ...
```

Figure S1: First part of KMC-dump application

```
    //-------------------------------------------------------------------------------
    // Open kmer database for listing and print kmers within min_count and max_count
    //-------------------------------------------------------------------------------

    if (!kmer_database.OpenForListing(input_file_name))
    {
      print_info();
      return EXIT_FAILURE ;
    }
    else
    {
      uint32 _kmer_length;
      uint32 _mode;
      uint32 _counter_size;
      uint32 _lut_prefix_length;
      uint32 _min_count;
      uint32 _max_count;
      uint64 _total_kmers;

      kmer_database.Info(_kmer_length, _mode, _counter_size, _lut_prefix_length, _min_count, _max_count, _total_kmers);

      float counter;
      std::string str;

      CKmerAPI kmer_object(_kmer_length);

      if(min_count_to_set)
        if (!(kmer_database.SetMinCount(min_count_to_set)))
          return EXIT_FAILURE;
      if(max_count_to_set)
        if (!(kmer_database.SetMaxCount(max_count_to_set)))
          return EXIT_FAILURE;

      while(kmer_database.ReadNextKmer(kmer_object, counter))
      {
        kmer_object.to_string(str);

        if(_mode)
          fprintf(out_file, "%s\t%f\n", str.c_str(), counter);
        else
          fprintf(out_file, "%s\t%d\n", str.c_str(), (int)counter);
      }

      fclose(out_file);
    }

    return EXIT_SUCCESS;
}

// -------------------------------------------------------------------------
// Print execution options
// -------------------------------------------------------------------------
void print_info(void)
{
    std::cout << "KMC␣dump␣ver.␣" << KMC_VER << "␣(" << KMC_DATE << ")\n";
    std::cout << "\nUsage:\nkmc_dump␣[options]␣<kmc_database>␣<output_file>\n";
    std::cout << "Parameters:\n";
    std::cout << "<kmc_database>␣-␣kmer_counter's␣output\n";
    std::cout << "Options:\n";
    std::cout << "-ci<value>␣-␣print␣k-mers␣appearing␣less␣than␣<value>␣times\n";
    std::cout << "-cx<value>␣-␣print␣k-mers␣appearing␣more␣of␣than␣<value>␣times\n";
};
```

Figure S2: Second part of KMC-dump application

4 Database format

The KMC application creates output files with two extensions:

- .kmc_pre — information on k-mer prefixes (plus some other data) are stored here,
- .kmc_suf — information on k-mer suffixes and the related counters are stored here.

All integers in the KMC output files are stored in LSB (least significant byte first) format.

4.1 The .kmc_pre file structure

A .kmc_pre file contains, in the order:

- [marker],
- [data],
- [header],
- [header position],
- [marker] (another copy, to signal the file is not truncated).

[marker]

4 bytes with the letters: KMCP.

[header position]

An integer consisting of the last 4 bytes in the file. Its contains the relative position of the beginning of the field [header]. After opening the file, one should do the following:

1. Read the first 4 bytes and check if they contain the letters KMCP.
2. Jump to position 4 bytes back from end of file and read the header position x.
3. Jump to position $x + 4$ bytes back from end of file and read the header.
4. Read [data].

[header]

The header contains fields describing the file .kmc_pre:

- uint32 kmer_length — k-mer length,
- uint32 mode — mode: 0 (occurrence count) or 1 (counting according to Quake quality),
- uint32 counter_size — counter field size: for mode 0 it is 1, 2, 3, or 4; for mode 1 it is always 4,
- uint32 lut_prefix_length — the length (in symbols) of the prefix cut off from k-mers; it is invariant of the scheme that 4 divides $kmer\_length - lut\_prefix\_length$,

- uint32 min_count — minimum number of k-mer occurrences to write in the database (if the counter is smaller, the k-mer data are not written),
- uint32 max_count — maximum number of k-mer occurrences to write in the database (if the counter is greater, the k-mer data are not written),
- uint64 total_kmers — total number of k-mers in the database,
- uint32 tmp[8] — not used in the current version.

[data]

Here are k-mer prefix data. More precisely, it is an array of uint64 elements, of size $4^{lut\_prefix\_length}$. Position x in the array stores the number of different k-mers whose prefix of length lut_prefix_length is (in binary) less than x. DNA symbols are encoded as follows: $A \leftarrow 0$, $C \leftarrow 1$, $G \leftarrow 2$, $T \leftarrow 3$. For example, if the queried k-mer is ACGACTGAT and $lut\_prefix\_length = 4$, then we cut off the first 4 symbols, i.e., the prefix ACGA, which is interpreted in binary as $x = 24$ (since $0 \times 2^6 + 1 \times 2^4 + 2 \times 2^2 + 0 \times 2^0 = 24$). Now we look into "data" at locations x and $x + 1$ to read, e.g., 1523 and 1685. This means that in the file .kmc_suf in records from 1523 to $1685 - 1$ there are suffixes of the k-mers with prefix ACGA. Having got this range, we can now binary search the suffix CTGAT.

4.2 The.kmc_suf file structure

A .kmc_suf file contains, in order:

- [marker],
- [data],
- [marker] (another copy, to signal the file is not truncated).

The k-mers are stored with their leftmost symbol first, packed into bytes. For example, CCACAAAT is represented as 0x51 (for CCAC), 0x03 (for AAAT). Integers are stored according to the LSB (little endian) convention, floats are stored in the same way as they are stored in the memory.

[marker]

4 bytes with the letters: KMCS.

[data]

An array record_t records[total_kmers].

total_kmers is a value taken from the .kmc_pre file.

record_t is a type representing a k-mer. Its first field is the k-mer suffix string, stored on $(kmer\_length - lut\_prefix\_length)/4$ bytes. The next field is $counter\_size$, with the number of bytes used by the counter, which is either a $1 \ldots 4$-byte integer, or a 4-byte float.

5 Experimental results

5.1 Test platforms

K-mer Counter (KMC), was implemented in C++11, using gcc compiler (version 4.7.1) for the linux build and Microsoft Visual Studio 2012 for the Windows build. Experiments comprise also the tools Jellyfish and BFCounter.

Two test machines were used. One was a 4 AMD Opteron^{TM}6136 2.4 GHz CPUs (32 cores) server with 128 GB RAM, and fast RAID-0 disk matrix of total size 2.0 TB. The other was a "home" PC, with 6-core AMD Phenom II 1090 3.2 GHz CPU, 16 GB RAM and 3 SATA HDD of sizes 2 TB each. The hard disks at the PC machine were: two Seagate Barracuda Green ST2000DL003 with 5,900 rpm and one Hitachi Deskstar 7K3000 with 7,200 rpm.

5.2 Parameters of programs

BFCounter

The main problem with running BFCounter (ver. 0.2) concerns proper estimation of the number of distinct k-mers. In the experiments we set this parameter to be about 20 percent greater than the actual number of distinct k-mers (which was known from a KMC test).

Jellyfish

Jellyfish (ver. 0.5) requires to give as a parameter the size of the hash table, which should be a bit larger than the total number of distinct k-mers. Similarly as for BFCounter we passed the value about 20 percent greater than the number of distinct k-mers (known from prior KMC execution). Unfortunately, for some cases (denoted by asterisk in the tables with experimental results), it resulted in Jellyfish crash due to the insufficient amount of RAM (128 GB of RAM was available in the test machine). In such cases we halved the parameter value and ran Jellyfish again, which produced two output files. These files should be merged then, but unfortunately, Jellyfish was unable to do that due to insufficient amount of RAM.

To allow right comparison with BFCounter (which does not count unique k-mers), we set in the execution of Jellyfish that it should not count k-mers with count value less than 2.0.

Probably due to some bug, when the output file name specified in the command line is shorter than 10 letters, Jellyfish often dramatically slows down (2–3 times). Thus, we always used long file names.

DSK

DSK (ver. 1.4993) was executed with default parameters that means 5 GB limit of RAM. The k-mers occurring less than 2 times were excluded.

KMC

KMC was executed with setting that k-mers occurring less than 2 times should not be counted. The parameter `-p` was set to 3 for two smallest data sets, to 4 for *Caenorhabditis elegans* data set and to 5 for two human data sets.

5.3 Results

The results presented at the following pages are for 5 data sets characterized in the main part of the paper. For 3 data sets examined in the main paper we duplicate here the tables and provide extra results (for other values of k).

Table S1: k-mers counting results for *D. ananassae* (8.7 GB FASTQ file or 6 gzipped FASTQ files of total size 1.8 GB). RAM and disk spaces are in GB (1GB=2^{30}B). Time is in seconds. The programs were used for the number of threads adjusted to the number of cores to achieve maximum speed.

Algorithm	$k=22$		$k=25$		$k=28$		$k=31$		$k=40$		$k=55$		$k=70$	
	Space	Time	Space	Time	Space	Time	Space	Time	Space	Time	Space	Time	Space	Time
**Classic counters**														
32-core server														
BFCounter	0.6/ 0	2,157	0.6/ 0	2,082	failed		failed		failed		failed		failed	
Jellyfish	10/ 0	42	10/ 0	48	11/ 0	44	11/ 0	44	—		—		—	
KMC	32/ 4	43	32/ 5	44	32/ 7	48	32/ 7	49	32/ 9	71	32/ 8	56	32/ 3	37
KMC	16/ 4	45	16/ 5	44	16/ 7	47	16/ 7	44	16/ 9	69	16/ 8	55	16/ 3	38
KMC^{gz}	32/ 4	47	32/ 5	45	32/ 7	49	32/ 7	50	32/ 9	72	32/ 8	59	32/ 3	39
KMC^{gz}	16/ 4	46	16/ 5	49	16/ 7	49	16/ 7	48	16/ 9	75	16/ 8	56	16/ 3	53
6-core PC														
KMC	11/ 4	103	11/ 5	130	11/ 6	135	11/ 7	143	11/ 8	180	11/ 8	164	11/ 3	108
KMC^{gz}	11/ 4	79	11/ 5	85	11/ 6	89	11/ 7	101	11/ 8	148	11/ 7	123	11/ 3	60
**Quake-compatible counters**														
32-core server														
BFCounter	0.7/ 0	2,126	0.7/ 0	2,049	0.7/ 0	1,889	0.7/ 0	1,850	0.6/ 0	1,513	0.5/ 0	1,046	0.3/ 0	425
Jellyfish	10/ 0	54	10/ 0	52	10/ 0	53	10/ 0	52	—		—		—	
KMC	32/12	77	32/12	78	32/13	91	32/14	82	32/14	95	32/11	77	32/ 4	40
KMC	16/12	61	16/12	61	16/13	62	16/14	67	16/14	76	16/11	63	16/ 4	39
KMC^{gz}	32/12	85	32/12	85	32/13	86	32/14	86	32/13	101	32/11	80	32/ 4	46
KMC^{gz}	16/12	94	16/12	65	16/13	67	16/14	70	16/13	92	16/11	77	16/ 4	57
6-core PC														
KMC	11/12	171	11/12	187	11/13	190	11/13	194	11/13	218	11/10	195	11/ 4	140
KMC^{gz}	11/12	173	11/12	177	11/13	171	11/13	178	11/13	214	11/10	172	11/ 4	78

Table S2: k-mers counting results for *Caenorhabditis elegans* (16.4 GB FASTQ file or 2 gzipped FASTQ files of total size 4.6 GB). Test methodology and column description are just as for Table S1.

	$k = 22$		$k = 25$		$k = 28$		$k = 31$		$k = 40$		$k = 55$		$k = 70$	
Algorithm	Space	Time	Space	Time	Space	Time	Space	Time	Space	Time	Space	Time	Space	Time
**Classic counters**														
32-core server														
BFCounter	4/ 0	10,407	4/ 0	10,142	failed		failed		failed		failed		failed	
Jellyfish	21/ 0	88	22/ 0	130	22/ 0	89	23/ 0	89	—		—		—	
KMC	32/10	105	32/14	99	32/18	115	32/20	123	32/27	185	32/31	191	32/28	204
KMC	16/11	93	16/14	84	16/18	93	16/21	105	16/27	163	16/32	160	16/28	175
KMC^{gz}	32/10	134	32/14	126	32/18	138	32/20	146	32/27	199	32/31	203	32/28	228
KMC^{gz}	16/11	119	16/14	127	16/18	121	16/21	126	16/27	195	16/32	182	16/29	218
6-core PC														
KMC	11/11	274	11/14	316	11/18	343	11/21	379	11/27	507	11/32	553	11/28	576
KMC^{gz}	11/11	233	11/14	267	11/18	333	11/21	359	11/27	514	11/32	542	11/29	567
**Quake-compatible counters**														
32-core server														
BFCounter	4/ 0	10,349	4/ 0	10,044	4/ 0	9,527	4/ 0	9,338	4/ 0	8,213	4/ 0	6,689	4/ 0	4,709
Jellyfish	24/ 0	143	25/ 0	154	25/ 0	132	26/ 0	148	—		—		—	
KMC	32/31	173	32/34	173	32/37	179	32/38	188	32/42	245	32/43	243	32/36	249
KMC	16/32	166	16/34	179	16/37	154	16/39	164	16/43	212	16/44	214	16/37	277
KMC^{gz}	32/31	184	32/34	186	32/37	188	32/38	197	32/43	247	32/43	249	32/36	262
KMC^{gz}	16/32	168	16/34	173	16/37	165	16/40	171	16/43	218	16/44	230	16/37	238
6-core PC														
KMC	11/32	562	11/34	581	11/37	635	11/40	678	11/43	750	11/44	784	11/37	745
KMC^{gz}	11/32	555	11/34	581	11/37	627	11/40	673	11/43	749	11/44	772	11/37	727

Table S3: k-mers counting results for *Zea mays* (45.9 GB FASTQ file or 108 gzipped FASTQ files of total size 16.3 GB). Test methodology and column description are just as for Table S1.

Algorithm	$k=22$ Space	$k=22$ Time	$k=25$ Space	$k=25$ Time	$k=28$ Space	$k=28$ Time	$k=31$ Space	$k=31$ Time	$k=40$ Space	$k=40$ Time	$k=55$ Space	$k=55$ Time	$k=70$ Space	$k=70$ Time
**Classic counters**														
32-core server														
BFCounter	12/ 0	38,050	13/ 0	37,509	failed		failed		failed		failed		failed	
Jellyfish	60/ 0	358	63/ 0	524	66/ 0	378	70/ 0	395	—		—		—	
KMC	32/ 40	328	32/ 56	346	32/ 72	433	32/ 82	471	32/125	792	32/183	1,082	32/230	1,576
KMC	16/ 42	287	16/ 56	302	16/ 74	385	16/ 87	455	16/125	785	16/189	1,107	16/235	1,608
KMC^{gz}	32/ 40	423	32/ 56	365	32/ 72	480	32/ 82	532	32/125	804	32/183	1,060	32/230	1,530
KMC^{gz}	16/ 41	338	16/ 56	310	16/ 74	347	16/ 86	424	16/125	736	16/188	1,037	16/235	1,566
6-core PC														
KMC	11/ 42	809	11/ 58	893	11/ 75	1,138	11/ 89	1,324	11/126	2,151	11/188	3,081	11/238	4,846
KMC^{gz}	11/ 42	734	11/ 58	889	11/ 75	1,180	11/ 89	1,405	11/126	2,160	11/188	3,227	11/238	4,846
**Quake-compatible counters**														
32-core server														
BFCounter	34/ 0	44,025	40/ 0	44,838	failed		failed		failed		failed		failed	
Jellyfish	72/ 0	727	75/ 0	662	78/ 0	726	82/ 0	741	—		—		—	
KMC	32/117	690	32/133	750	32/150	824	32/160	888	32/198	1,189	32/252	1,479	32/298	2,048
KMC	16/121	681	16/135	757	16/150	782	16/165	902	16/198	1,213	16/257	1,530	16/303	2,166
KMC^{gz}	32/117	675	32/133	711	32/150	787	32/160	850	32/198	1,142	32/252	1,422	32/297	1,989
KMC^{gz}	16/121	666	16/135	698	16/150	801	16/164	837	16/198	1,144	16/257	1,479	16/303	2,222
6-core PC														
KMC	11/123	1,866	11/134	2,125	11/150	2,370	11/167	2,532	11/198	3,360	11/256	4,574	11/304	6,327
KMC^{gz}	11/123	1,951	11/135	2,203	11/150	2,346	11/167	2,658	11/198	3,611	11/256	4,558	11/304	6,496

Table S4: k-mers counting results for *Homo sapiens* NA19238 individual (353 GB FASTQ file or 463 gzipped FASTQ files of total size 116.6 GB). Test methodology and column description are just as for Table S1. The asterisk signs (for Jellyfish) denote that two separate databases were constructed by Jellyfish due to the memory limit of the machine (128 GB RAM) and Jellyfish reported that to merge these databases it needs more RAM, so these times are underestimated.

	$k = 22$		$k = 25$		$k = 28$		$k = 31$	
Algorithm	Space	Time	Space	Time	Space	Time	Space	Time
**Classic counters**								
32-core server								
BFCounter	46/ 0	114,083	41/ 0	99,468	failed		failed	
Jellyfish	50/ 0	2,303	64/ 0	2,258	75/ 0	2,208	87/ 0	2,107
KMC	32/104	1,405	32/130	1,488	32/133	1,522	32/121	1,471
KMC	16/107	1,548	16/131	1,657	16/141	1,684	16/128	1,568
KMC^{gz}	32/104	1,040	32/129	1,066	32/132	1,055	32/120	989
KMC^{gz}	16/107	1,278	16/130	1,631	16/141	1,662	16/125	1,307
6-core PC								
KMC	11/107	3,482	11/128	3,442	11/138	3,584	11/127	3,515
KMC^{gz}	11/107	2,198	11/128	2,206	11/138	2,303	11/127	2,365
**Quake-compatible counters**								
32-core server								
BFCounter	70/ 0	171,888	72/ 0	180,861	failed		failed	
Jellyfish	100/ 0	4,339	57/230	2,891*	64/192	3,246*	70/210	3,161*
KMC	32/311	2,585	32/302	2,467	32/282	2,347	32/237	2,106
KMC	16/315	2,615	16/305	2,730	16/283	2,592	16/245	2,284
KMC^{gz}	32/310	2,071	32/302	1,995	32/282	1,880	32/237	1,690
KMC^{gz}	16/318	2,273	16/304	2,611	16/283	2,015	16/244	2,707
6-core PC								
KMC	11/316	5,538	11/298	5,533	11/277	5,184	11/242	5,016
KMC^{gz}	11/316	5,370	11/298	5,060	11/277	4,708	11/243	4,643

Table S5: k-mers counting results for *Homo sapiens* HG02057 individual (208 GB FASTQ file or 6 gzipped FASTQ files of total size 65.9 GB). Test methodology and column description are just as for Table S1. The asterisk signs (for Jellyfish) denote that two separate databases were constructed by Jellyfish due to the memory limit of the machine (128 GB RAM) and Jellyfish reported that to merge these databases it needs more RAM, so these times are underestimated.

	$k = 22$		$k = 25$		$k = 28$		$k = 31$		$k = 40$		$k = 55$		$k = 70$	
Algorithm	Space	Time	Space	Time	Space	Time	Space	Time	Space	Time	Space	Time	Space	Time
**Classic counters**														
32-core server														
Jellyfish	27/ 0	1,375	33/ 0	1,754	75/ 0	1,433	86/ 0	1,462	—		—		—	
KMC	32/130	1,221	32/184	1,486	32/220	1,706	32/244	1,826	32/341	2,486	32/391	2,722	32/354	2,778
KMC	16/134	1,376	16/185	1,592	16/234	1,872	16/259	1,997	16/343	2,664	16/405	2,967	16/366	2,989
KMC^{gz}	32/130	1,249	32/184	1,318	32/219	1,505	32/244	1,682	32/342	2,304	32/391	2,597	32/354	2,611
KMC^{gz}	16/134	1,195	16/185	1,564	16/234	1,732	16/258	1,836	16/343	2,479	16/403	2,909	16/363	2,772
6-core PC														
KMC	11/137	2,939	11/186	3,280	11/234	3,782	11/263	4,312	11/343	6,133	11/405	7,770	11/363	8,145
KMC^{gz}	11/136	2,623	11/186	3,242	11/235	4,041	11/261	4,541	11/343	6,306	11/405	7,020	11/363	7,765
**Quake-compatible counters**														
32-core server														
Jellyfish	51/ 0	2,426	57/ 0	2,300	59/126	2,503*	70/139	3,192*	—		—		—	
KMC	32/388	2,612	32/428	2,805	32/468	3,011	32/480	3,119	32/537	3,541	32/542	3,546	32/456	3,515
KMC	16/402	2,990	16/432	3,122	16/468	3,405	16/499	3,579	16/539	4,300	16/552	4,175	16/470	4,426
KMC^{gz}	32/387	2,409	32/428	2,640	32/468	2,860	32/480	2,988	32/537	3,370	32/536	3,357	32/456	3,181
KMC^{gz}	16/400	2,760	16/431	2,957	16/468	3,285	16/497	3,351	16/498	4,083	16/552	4,038	16/467	3,724
6-core PC														
KMC	11/404	6,625	11/433	7,123	11/469	7,741	11/502	8,252	11/539	9,673	11/552	11,135	11/465	9,988
KMC^{gz}	11/403	6,783	11/431	7,463	11/468	8,034	11/503	8,345	11/539	9,764	11/553	9,775	11/466	9,410

6 Automatic setting of parameters in KMC

The automatic setting of parameters mechanism tries to allocate the available resources (i.e., CPU cores) in the best possible way. The optimal number of threads for the parts of the algorithm is, however, hard to obtain, since it depends on many things, like the compression method of input files, the number and speed of disks, etc. Thus, our automatic mechanism is obviously suboptimal, nevertheless, experiments show that it performs reasonably well. If the results are unsatisfactory, the KMC user can specify these parameters from command line.

The most important factor of the mechanism is the number of available cores (possibly overridden if the user specifies it with `-s?` parameters).

Table S6: Automatic selection of internal parameters in KMC. T is the number of cores, D the number of working directories, F the number of input files

FASTQ readers<br>N_{F}	Splitters<br>N_{Sp}	Compactors<br>N_{C}	Disk writers and Bin writers<br>N_{D}	Sorters<br>N_{So}	Sorting threads per sorter<br>N_{St}
**Small PC:** $T < 6$					
$\min(F, T)$	$\max(1, T-1)$	$\max(1, T-1)$	D	1	T
**Large PC:** $6 \le T \le 12$					
$\min(F, T)$	$\max(1, T-1)$	$\max(1, T-1)$	D	2	$T/2+1$
**Small server:** $12 < T \le 24$					
$\min(F, T/3)$	$(T-N_{\mathrm{F}})/2$	$T-N_{\mathrm{F}}-N_{\mathrm{Sp}}-1$	D	$T/6$	T/N_{So}
**Large server:** $24 < T$					
$\min(F, T/3)$	$(T-N_{\mathrm{F}})/2$	$T-N_{\mathrm{F}}-N_{\mathrm{Sp}}-1$	D	$T/8$	T/N_{So}

7 Selected components of the KMC algorithm (codes not shown in the main part of the paper)

FASTQ-READER(*FL_queue*)

Input: *FL_queue — queue with list of input file names*

while (*file_name* ← *FL_Queue*.pop()) **not** empty **do**
    file ← Open file *file_name*
    while (*block* ← *file*.read_next_block()) **not** empty **do**
        FASTQ_parts_queue.push(*block*)
    Close *file*

Figure S3: Reading FASTQ/FASTA files. Each block, of several megabytes, is rounded to the nearest complete read. The blocks are inserted into a FASTQ parts queue.

COMPACTOR(...)

Input: ... — *queue with parts of FASTQ/FASTA files*

while (*bin_id*, *bin_package* ← *Bin_part_packages_queue*.pop()) **not** empty **and**
    not *Bin_part_packages_queue*.finished() **do**
    if *bin_package* **not** empty **then**
        Try to compact *bin_package* on $p_2 + 4$ symbols
        Put (possibly compacted) *bin_package* to *Compacted_buffers_queue*

Figure S4: Algorithm of extra compacting bin packages prior to writing to disk. The output of this stage are compacted packages.

DISK-WRITER(...)

Input: ... — *queue with parts of FASTQ/FASTA files*

while (*bin_id*, *buffer* ← *Compacted_buffer_queue*.pop()) **not** empty **and**
    not *Compacted_buffer_queue*.finished() **do**
    if *buffer* **not** empty **then**
        Write *buffer* to disk file related with *bin_id*

Figure S5: Disk-writer algorithm

BIN READER(...)

Input: ... — *queue with parts of FASTQ/FASTA files*

while (*bin_id* ← *Bin_ids_queue*.pop()) **not** empty **do**
    bin_data ← read whole file related to *bin_id*
    Bins_queue.push(*bin_id, bin_data*)

Figure S6: Bin reader algorithm

COMPLETER(...)

Input: ... — *queue with parts of FASTQ/FASTA files*

while (*bin_id, buffers* ← *Sorted_and_compacted_bins_priority_queue*.pop()) **not** empty **and**
    not *Sorted_and_compacted_bins_priority_queue*.finished() **do**
    if *buffers* **not** empty **then**
        Store *buffers* to two output files

Figure S7: Completer algorithm
